# Organizational Changes of the Daughter Basal Complex during the Parasite Replication of Toxoplasma gondii

**DOI:** 10.1371/journal.ppat.0040010

**Published:** 2008-01-18

**Authors:** Ke Hu

**Affiliations:** Department of Biology, Indiana University, Bloomington, Indiana, United States of America; University of Michigan, United States of America

## Abstract

The apicomplexans are a large group of parasitic protozoa, many of which are important human and animal pathogens, including Plasmodium falciparum and Toxoplasma gondii. These parasites cause disease only when they replicate, and their replication is critically dependent on the proper assembly of the parasite cytoskeletons during cell division. In addition to their importance in pathogenesis, the apicomplexan parasite cytoskeletons are spectacular structures. Therefore, understanding the cytoskeletal biogenesis of these parasites is important not only for parasitology but also of general interest to broader cell biology. Previously, we found that the basal end of T. gondii contains a novel cytoskeletal assembly, the basal complex, a cytoskeletal compartment constructed in concert with the daughter cortical cytoskeleton during cell division. This study focuses on key events during the biogenesis of the basal complex using high resolution light microscopy, and reveals that daughter basal complexes are established around the duplicated centrioles independently of the structural integrity of the daughter cortical cytoskeleton, and that they are dynamic “caps” at the growing ends of the daughters. Compartmentation and polarization of the basal complex is first revealed at a late stage of cell division upon the recruitment of an EF-hand containing calcium binding protein, TgCentrin2. This correlates with the constriction of the basal complex, a process that can be artificially induced by increasing cellular calcium concentration. The basal complex is therefore likely to be a new kind of centrin-based contractile apparatus.

## Author Summary

Toxoplasma gondii is one of the most prevalent parasites in warm-blooded animals and a highly important human pathogen. It is the most common cause of congenital neurological defects in humans and also causes devastating opportunistic infections in immuno-compromised patients. Many of its 5,000 relatives in phylum Apicomplexa are also important human or animal pathogens, including Plasmodium sps, which kill more than a million people every year. The pathogenesis of the diseases that these parasites cause absolutely depend on their ability to replicate, which in turn completely depends on the proper assembly of the parasite cytoskeletons. Here I probe how the basal complex, a novel cytoskeletal compartment contained within the basal end of T. gondii, is assembled during daughter cell formation of this parasite. I found that the daughter basal complex is one of the first cytoskeletal structures assembled during T. gondii cell division. In addition, the basal complex is likely to be a new kind of centrin-based contractile apparatus, as its polarization is first revealed upon the recruitment of a calcium binding protein, TgCentrin2, which correlates with the constriction of the basal complex, a process that can be artificially induced by increasing cellular calcium concentration.

## Introduction

The phylum Apicomplexa contains ∼5,000 species of obligate intracellular protozoan parasites, many of which are important human or animal pathogens. Toxoplasma gondii is the most common cause of congenital neurological defects in humans, and an agent for devastating opportunistic infections in immunocompromised patients. Plasmodium falciparum, the most lethal form of malaria, kills more than a million people every year.

The pathogenesis of the diseases that these parasites cause absolutely depend on their ability to replicate. In the absence of massive, uncontrolled expansion of the parasite population, the infections are benign. Thus, the understanding of parasite growth and division is crucial for developing effective therapies. The apicomplexan parasite cytoskeletons provide the framework for organellar replication and partition, and are essential for parasite survival and replication, therefore are attractive potential drug targets [1–3]. In addition to their importance in pathogenesis, the apicomplexan parasite cytoskeletons are marvelous structures [4–7]. Thus understanding the cytoskeletal biogenesis of these parasites is also of general interest for cell biology.

In spite of the diversity in their host species, host selectivity, and the diseases they cause, the apicomplexan parasites share similar basic cell biology. In particular, all apicomplexans are obligate intracellular parasites. Most of them replicate inside a specialized parasitophorous vacuole within the host cell in an unusual process where the daughter cytoskeletons preform *de novo* prior to cytokinesis [1–3,8].

The daughter cytoskeletons contain several distinct sets of microtubules that are likely involved in different aspects of parasite motility and division [7,9,10]; the Inner Membrane Complex (IMC), formed of flattened vesicles with a regular intramembranous particle lattice likely associated with the cortical microtubules [5,11–13]; a set of proteins weakly homologous to intermediate filaments underlying the IMC [2,14]; and a cytoskeletal apical complex that is closely associated with the membrane-bound invasion organelles [6,15,16]. During parasite division, the membrane bound organelles (including the nucleus, ER, Golgi apparatus, mitochondrion, apicoplast and a collection of secretory organelles such as rhoptries and micronemes) will be replicated and partitioned into the growing daughter cytoskeletons, until the end of the cytokinesis when the daughters bud out of the mother and take over mother's plasma membrane [1,2].

Compared with other compartments of the parasite body, much less was known about the architecture of the basal end of the parasite. We and others found that the basal end of the parasite contains a specialized compartment with distinct morphology and molecular composition [16,17]. It is constructed together with the rest of the daughter cytoskeleton, first as ring-like structures capping the growing ends of the daughters, which then constrict and eventually cap the basal pole of the adult parasite. Because of its distinct localization, organization and molecular composition, we used the term “basal complex” to represent this distinct compartment [16].

**Figure 1:**
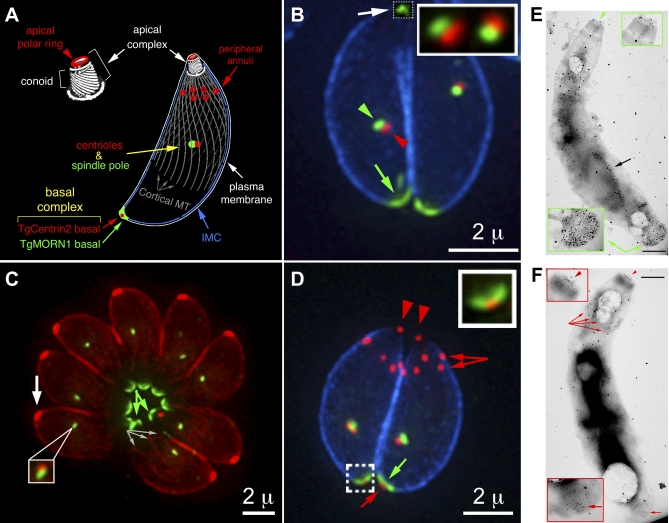
The Distribution of Two Basal Complex Components, TgMORN1 and TgCentrin2, in Mature Parasites (A) The major structural reference points of the T. gondii cytoskeleton referred to throughout this paper are illustrated in the cartoon drawing of an interphase adult parasite. The cytoskeleton of T. gondii includes the apical and the basal complexes, the spindle pole, the centriole, and the cortical cytoskeleton, which includes all of the cytoskeletal elements aligning the parasite body (i.e., cortical microtubules, the IMC, and the filamentous network underlying the IMC), except for the apical and basal complexes. (B) In interphase parasites, TgMORN1 (green, EGFP-TgMORN1) forms a cap at the extreme basal end of the parasite (green arrow), filling the gap at the basal end of the IMC (blue, labeled by anti-IMC1 monoclonal antibody plus Alexa350-anti-mouse IgG). TgMORN1 also weakly concentrates in the apical complex (dotted square indicated by the white arrow. Stronger contrast enhancement of TgMORN1 labeling is applied to this region to highlight the apical complex labeling of TgMORN1). TgMORN1 is also localized to the spindle pole (green arrowhead), which is juxtaposed to the centriole (red, mCherryFP-TgCentrin1, red arrowhead) during interphase. (C) In interphase parasites, the basal labeling of TgMORN1 (green arrows) is clearly separated from the cortical microtubules (red, mCherryFP-TgTubA1, small gray arrows). The inset shows a magnified view of the centriole/spindle pole assembly, showing red TgTubA1 in the centriole and green TgMORN1 in the spindle pole. At this point in the cell cycle, tubulin labeling of the spindle pole is weak, but will become stronger in early cell division (cf. Figure 5). White arrow, conoid labeling by mCherryFP-TgTubA1. (D) TgMORN1 and TgCentrin2 occupy different subcompartments within the basal complex, with the TgCentrin2 compartment (red arrow at the basal) located posterior to the TgMORN1 basal labeling (green arrow). These parasites are at the very end of interphase, as indicated by the recent duplication of the centriole in the parasite on the left, but not in the parasite on the right. Green, mCherryFP-TgMORN1 (pseudo-color, for consistency in the color scheme); red, EGFP-TgCentrin1 (pseudo-color); blue, anti-IMC1 antibody detected by Alexa350-anti-mouse IgG. Red arrowheads, TgCentrin2 labeling of the apical polar ring (cf. [A], [16]). Red arrows near the apical portion of the parasite apical, TgCentrin2 peripheral annuli (cf. [A], [16]). Insets are at 2× magnification. The insets in (B) and (D) do not include the anti-IMC1 labeling in order to emphasize the differences in localization betweenTgMORN1/ TgCentrin1 (B) and TgMORN1/TgCentrin2 (D) labeling. (E) An EM image of an EGFP-TgMORN1 transgenic parasite extracted with 0.5% TritonX-100, immunogold labeled with anti-GFP antibody and negatively stained with phosphotungstic acid. Numerous gold particles are located in the basal complex (bottom inset, green arrows). There is also a small concentration of gold particles in the apical polar ring (top inset, green arrowhead). The cluster of gold particles aligned together (black arrow) at the bottom half of the parasite body is likely to be the labeling of an EGFP-TgMORN1 fiber sometimes seen to form in extracellular parasites [16]. (F) An immuno-EM image of an EGFP-TgCentrin2 transgenic parasite, extracted, labeled, and stained as described in (E). As previously reported [16], there are clear concentrations of EGFP-TgCentrin2 labeling in the apical polar ring (top inset, red arrowheads) and several peripheral annuli (red arrows). Consistent with the light microscopy data, the basal complex labeling of EGFP-TgCentrin2 is considerably lighter than that of EGFP-TgMORN1 (bottom inset, red arrows). A concentration of gold particles within a ∼250 nm patch at the extreme basal end of the parasite is often seen, which likely corresponds to the concentration of TgCentrin2 basal labeling at the light microscopy level (cf. [D]). Both TgMORN1 and TgCentrin2 display certain levels of localization along the parasite body, which is likely to be from the proteins in the cytoplasmic pool. Scale bars = 500 nm; Insets are at 1.5× magnification.

In adult parasites, the basal complex occupies the basal gap of the IMC, which, together with the underlying filamentous network, encloses the entire parasite body except for the extreme apical and basal ends (Figure 1A and 1B) (the gap at the apical end of IMC is occupied by the cytoskeletal apical complex, an intricate assembly that includes the conoid, a tubulin-based molecular machine that does not utilize conventional microtubules; three polar rings; and two intra-conoid microtubules [Figure 1A and 1C] [6,7]). The basal complex is separated by more than 1.5 μm from another set of major cytoskeletal elements, the cortical microtubules, which emanate from the most posterior of the three polar rings and extend ∼2/3 of the length of the parasite body (Figure 1A and 1C).

The basal complex contains several distinguishable regions organized along its anterior-posterior axis, and is composed of substructures defined by different protein markers, including TgMORN1, TgCentrin2 and TgDLC, a dynein light chain of T. gondii [16]. Interestingly, these basal complex proteins are also components of the apical complex and the centriole/spindle pole assembly (Figure 1A–1D). The similarity in protein composition among these structurally and spatially distinct cytoskeletal assemblies is particularly intriguing given the *de novo* nature of the construction of the apical and the basal complex, because the centriole/spindle pole assembly are the only structures inherited by the daughter parasites during cell division, and the centrioles are the only cytoskeletal structure in T. gondii that can self-replicate, thus capable of propagating the structural information for building a new cytoskeleton to the daughters. Much of this study therefore focuses on the spatial-temporal coordination among the origination of the basal complex, the duplication of the centriole/spindle pole assembly, and the construction of the daughter cortical cytoskeleton. (Throughout this paper, “the cortical cytoskeleton” is used to refer to the entire framework of cytoskeleton elements that aligns the parasite body [i.e. cortical microtubules, the IMC and the filamentous network underlying the IMC] except for the apical and the basal complexes [Figure 1A]).

The basal complex is a new defining feature of T. gondii, and likely to be conserved in other important apicomplexan parasites [16,17]. To understand how this compartment comes into being and develops with the rest of the daughter cytoskeleton is crucial for elucidating how polarity is established in these parasites. It should be noted that the daughter cytoskeletons are built afresh, and the mother's body axis is apparently not used in the polarity determination of the daughters, since each daughter axis is more or less randomly oriented with respect to the mother and the other daughter [1,2].

Due to the limited spatial and temporal resolution of previous studies, and the fact that appropriate specific markers were not known, the precise sites and timing of basal complex initiation and maturation are not known. This study focuses on the key organizational changes of the daughter basal complex with respect to the centriole replication cycle as well as the maturation of the daughter parasite cortical cytoskeleton using high resolution wide-field deconvolution light microscopy. It reveals that the daughter basal complex is initiated in the vicinity of the centrioles before the establishment of the daughter cortical cytoskeleton, and that its initiation and construction are likely to be independent of the structural integrity of the daughter cortical cytoskeleton. The constriction of the basal complex that begins at a late stage of cell division correlates with the recruitment of a typical EF-hand containing calcium binding protein, TgCentrin2, to its posterior compartment, which reveals the compartmentation and polarity of the basal complex in the developing daughter. Further, this constriction can be artificially induced by elevated intracellular calcium concentration. The timing of the recruitment, the localization, and the molecular characteristics of TgCentrin2 make it the most plausible candidate for driving the constriction of the basal complex during its maturation, and the basal complex in T. gondii is likely to be a new kind of centrin-based contractile apparatus.

## Results

### The Distribution of Two Basal Complex Components, TgMORN1 and TgCentrin2, in Mature Parasites

Two previously identified basal complex components in T. gondii, TgMORN1 and TgCentrin2 [16], occupy two distinct compartments in the basal complex of mature parasites. The TgMORN1 compartment is shaped into a cone that forms the main body of the basal complex (Figure 1A–1E), whereas TgCentrin2 is concentrated at the posterior tip of the basal complex (Figure 1D and 1F). Both TgCentrin2 and TgMORN1 are also components of the apical complex. In addition, they are localized to the spindle pole and the centrioles, respectively. The spindle pole and the centrioles are juxtaposed to each other during interphase (Figure 1B–1D), but well separated after the daughter cortical cytoskeletons have formed in the mother [7,16,18,19] (The slight displacement between the spindle pole and the centrioles in each interphase cell is a true spatial displacement, not an artifact of mis-registration induced by lens chromatic aberration or optical misalignment between the GFP and mCherryFP filters, because the shift between the red and green fluorescence of a multi-color 0.2 μm bead is clearly much smaller and below the resolution limit of the microscope [Figure S1]).

### Initiation and Construction of the Daughter Basal Complex at the Beginning of Cell Division

**Figure 2:**
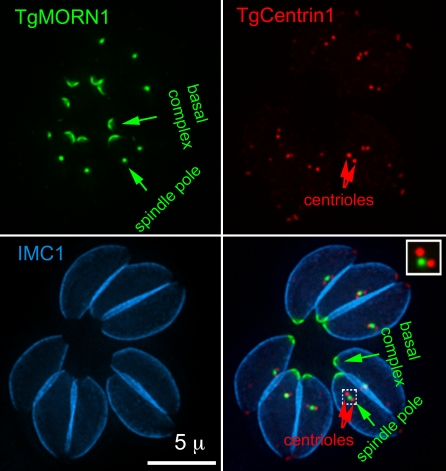
Centriole Duplication Precedes the Construction of the Basal Complex and the Separation of the Spindle Poles Images show a parasitophorous vacuole containing parasites whose centrioles have duplicated, but the spindle pole still appears as one single spot. No TgMORN1 labeling is seen around the centrosomal area other than in the spindle pole itself. In the three parasites at the bottom of the figure, the duplicated centrioles are still near the basal end of the nucleus, whereas in the other five, the centrioles have started or completed their return migration to the apical end of the nucleus. Green, EGFP-TgMORN1; red, mCherryFP-TgCentrin1; blue, anti-IMC1 antibody detected by Alexa350-anti-mouse IgG. Inset: 2× magnification of the region indicated by the dotted frame. The inset does not include the anti-IMC1 labeling in order to emphasize the difference in localization between TgMORN1 and TgCentrin1 in the centriole/spindle pole assembly. All images are maximum intensity projections of deconvolved 3D stacks.

**Figure 3:**
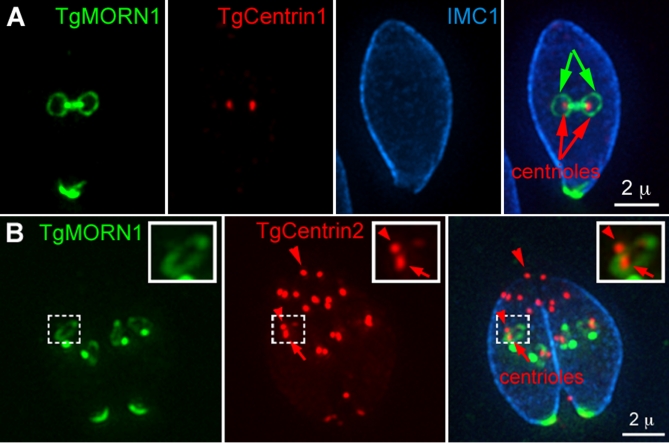
TgMORN1 Containing Ring Structures Are Found around the Centrioles after Centriole Duplication (A) Ring like structures containing TgMORN1 (green, EGFP-TgMORN1, green arrows) are constructed around the duplicated centrioles (red, mCherryFP-TgCentrin1, red arrows) after the duplicated centrioles return to the apical end of the nucleus. (B) TgCentrin2 (pseudo-colored red, EGFP-TgCentrin2) is undetectable in these TgMORN1 rings (pseudo-colored green, mCherryFP-TgMORN1). Arrows, centriole labeling by TgCentrin2; arrowheads, the mother and the future daughter's apical polar ring labeling by TgCentrin2. Note that a weak concentration of TgCentrin2 fluorescence can be seen at the upper-right portion of the inset in the TgCentrin2 panel. The identity of this concentration is not clear at the present. Blue, anti-IMC1 antibody detected by Alexa350-anti-mouse IgG. Lack of IMC1 antibody labeling in nascent daughters at this stage might be caused by poor epitope accessibility (cf. Figure 4 and Figure S3). Insets: 2× magnification of regions indicated by the dotted frames. All images are maximum intensity projections of deconvolved 3D stacks.

**Figure 4:**
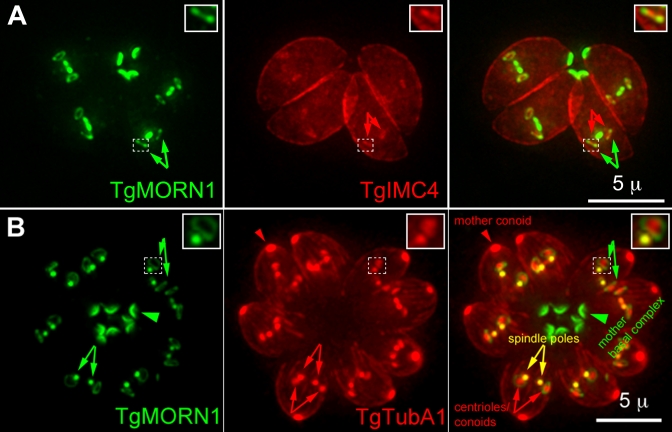
TgMORN1 Rings Are Formed before the Extension of the Daughter Cortical Cytoskeleton (A) A parasitophorous vacuole with parasites at similar stage as those in Figure 3 expressing mCherryFP-TgMORN1 (pseudo-colored green) and EGFP-TgIMC4 (pseudo-colored red). Faint planar concentrations of IMC (red arrows) are surrounded by the TgMORN1 rings (green arrows). (B) Eight parasites in one parasitophorous vacuole expressing EGFP-TgMORN1 (green) and mCherryFP-TubA1 (red). The EGFP-TgMORN1 rings (green arrows close to the dotted frames in the TgMORN1 and the merged panels) are formed around the centrioles and possibly nascent conoids highlighted by mCherryFP-TgTubA1 (red arrows at the bottom of the TgTubA1 and the merged panels). Both TgMORN1 and TgTubA1 also highlight the spindle poles, indicated by yellow arrows in the merged panel and the corresponding green and red arrows in the TgMORN1 and TgTubA1 panels. Green arrowheads, mother basal complex; red arrowheads, mother conoid. Insets: 2× magnification of regions indicated by the dotted frames. All images are maximum intensity projections of deconvolved 3D stacks.

The first sign of cell division is the migration of the centriole to the basal pole of the nucleus, where it replicates (Nishi M, Hu K, Murray J, Roos D, manuscript submitted). The replicated centrioles sandwich the spindle pole (Figure 2), which at this point still appears as one spot. Surprisingly, ring structures containing TgMORN1 are observed forming around the duplicated centrioles even before the separation of the future apical and basal regions of the daughter parasites (Figure 3). These rings are at the outer edges of the initially planar aggregations of daughter cytoskeletal elements that will later become the daughter cortical cytoskeletons (Figure 4). The TgMORN1 rings are therefore likely to be the precursor of the future daughter basal ring complex. Two other components of the mature basal complex in adult parasites, TgCentrin2 (Figure 3B) and TgDLC (data not shown), however, are not found in these early ring structures.

**Figure 5:**
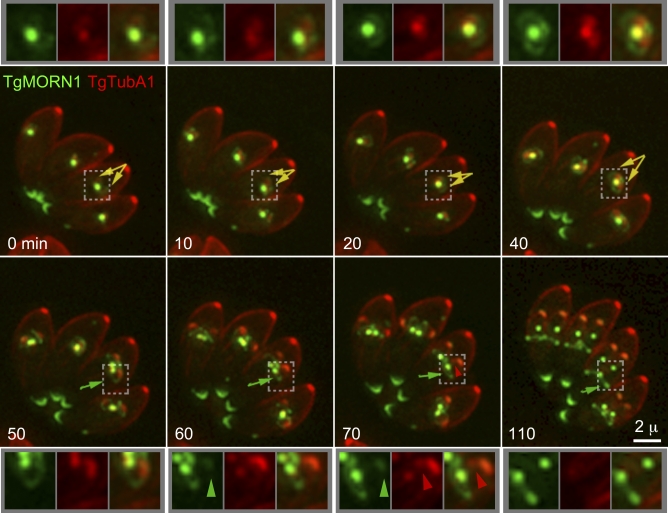
Time-lapse Images Showing That the TgMORN1 Ring Is Initiated around the Duplicated Centrioles (cf. Video S1) Images are selected time points from a time-lapse experiment tracking the cell division of four parasites expressing EGFP-TgMORN1 (green) and mCherryFP-TgTubA1 (red) (cf. Video S1). See text for detailed description of the time sequence. Top and bottom panels are 2× magnification of regions indicated by the dotted frames. Yellow arrows (t = 0–40 min), the initiation sites of the basal complex around the centrioles; green arrows (t = 50–110 min), daughter basal ring complex; green arrowheads (t = 60–70 min), TgMORN1 labeling of the daughter apical complex; red arrowheads (t = 70 min), the centriole labeling that is just separated from the conoid labeling of mCherryFP-TgTubA1. All images are maximum intensity projections of deconvolved 3D stacks.

How do these ring structures originate? Will they really become the basal complex in the daughters? To address these questions, I followed TgMORN1 distribution together with the daughter cortical cytoskeleton construction and centriole duplication in live parasites expressing EGFP-TgMORN1 and mCherryFP-Tg α1-tubulin (TgTubA1) (Figure 5; Video S1). The TgMORN1 ring first appears as small extra masses outside the spindle pole, and is located close to the recently duplicated centrioles, which lie on each side of the spindle poles (t = 10 min). 20–30 min later (t = 30–40 min), the fluorescence of mCherryFP-TgTubA1 in the centriole spot increases, likely correlated with the initial assembly of the conoid in the apical complex, of which TgTubA1 is a major component. At this point, the ring-like nature of the TgMORN1 containing structure becomes apparent, and it is centered around the centriole/conoid mass (t = 40 min). At t = 60 min, the centriole/conoid assemblies move apically above the plane of the TgMORN1 ring with the extension of the cortical microtubules, and the recruitment of TgMORN1 to the apical complex becomes clear. At this point the future apical and basal complexes are separated far enough to make it clear that the TgMORN1 rings formed at the beginning of the cell division are indeed precursors of the daughter basal complexes. The TgMORN1 rings remain at the basal ends of the daughter cortical cytoskeletons as the daughters grow (t = 70–110 min).

**Figure 6:**
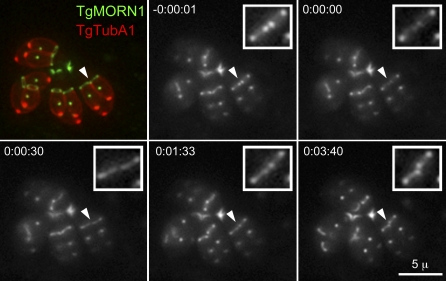
The Basal Complex Is a Dynamic “Cap” Time-lapse experiment tracking the exchange of EGFP-TgMORN1 in the basal complexes of growing daughters using Fluorescence Recovery After Photo-bleaching (FRAP). The fluorescence of EGFP-TgMORN1 in the basal complexes of two daughters was partially bleached (arrowheads, insets) at time 0, and the recovery was well underway at ∼90 s. Insets: 2× magnification of regions indicated by the arrowheads. The merged image of EGFP-TgMORN1 (green) and mCherryFP-TgTubA1 (red) is the maximum intensity projection of a deconvolved 3D stack. All the gray scale images are non-deconvolved single optical planes.

How is the basal complex able to remain at the constantly growing ends of the daughter cortical cytoskeletons? Studies in mammalian cells have shown that microtubule plus-end (MT-plus-end) binding proteins probably maintain their position at the growing ends of microtubules by rapid association and dissociation [20]. Fluorescence Recovery After Photobleaching (FRAP) analysis of daughter basal complexes in T. gondii reveals constant protein exchange between the daughter basal complex and the cytoplasm (Figure 6). Although it is difficult to calculate an exact t1/2 for the fluorescence recovery because of the noise introduced by constant fluctuation of the basal complex position due to daughter cell movement, the recovery is clearly underway by ∼90 sec after photobleaching. This result indicates that the basal complex is intrinsically a dynamic “cap”, thus suggesting a mechanism similar to microtubule association of MT-plus-end binding protein is possibly involved in retaining the basal complex at the growing ends of the daughter cortical cytoskeletons. However, the growth of the daughter cortical cytoskeleton is likely not to be the pre-requisite for the protein exchange in the basal complex, as the fluorescence in the mature basal complex also partially recovers after photobleaching (Figure S2).

### Is the Structural Integrity of the Daughter Cortical Cytoskeleton Required for the Initiation and Construction of the Daughter Basal Complex?

**Figure 7:**
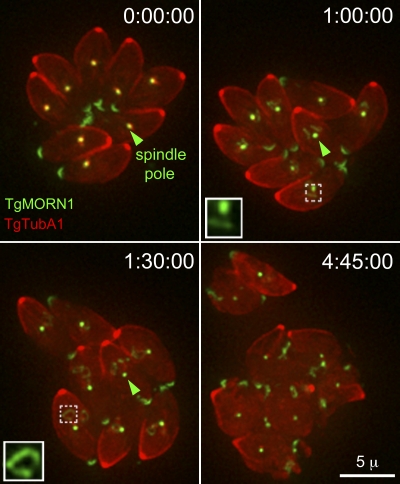
The Initial Construction of the TgMORN1 Ring Is Likely to Be Independent of the Structural Integrity of the Daughter Cortical Cytoskeleton (cf. Video S2) A parasitophorous vacuole containing eight dividing parasites expressing EGFP-TgMORN1 (green) and mCherryFP-TgTubA1 (red) was treated with 2.5 μM oryzalin at time 0. No daughter cortical microtubules can be detected in these parasites, but the initiation (cf. Video S2, 33–48 min) and the construction (cf. Video S2, 60–120 min) of the basal complex are not affected. As previously reported [9,17], the spindle pole (arrowheads) fails to replicate in the presence of 2.5 μM oryzalin. Insets: 2.5× magnification of regions indicated by the dotted frames. All images are maximum intensity projections of deconvolved 3D stacks.

The daughter cortical cytoskeleton and the basal complex appear to grow in concert during cell division. Is the construction and growth of the basal complex, a seemingly “downstream” structure, dependent on the structural integrity of the daughter cortical cytoskeleton? To address this question, the construction of the basal complex was tracked in living parasites whose cortical microtubule extension and the daughter cortical cytoskeleton formation were severely disrupted by treating with oryzalin, a plant herbicide that binds to T. gondii α-tubulin and inhibits the construction of the spindle and the cortical microtubules, but not the centriole replication, during daughter formation (Figure 7; Video S2) [9,10,21]. As expected, the mother's conoid, cortical microtubules, and basal complex are not affected by the oryzalin treatment, and the overall morphology of the mother cell remains normal until the distorted daughters attempt to bud. Daughter cortical microtubules, however, completely fail to appear. Despite the inhibition of the formation of functional daughter cortical cytoskeleton, the initiation of TgMORN1 ring formation proceeds normally (Video S2, 33 and 48 min). Furthermore, complete TgMORN1 rings are formed ∼30 min after the initiation (Video S2; Figure 7, t = 60 min), enlarge to ∼1.2 μm (similar to the diameter of the basal complex in untreated daughters with extending cortical cytoskeletons [cf. Figure 5, t = 70 min]), and retain their ring morphology till “budding”, at which point the organization of the basal complex becomes unclear due to the distorted parasite morphology. The initiation, construction and maintenance of the daughter basal ring complex are therefore independent of the structural integrity of the daughter cortical cytoskeleton.

### The Basal Complex Is Likely to Be a New Kind of Centrin-based Contractile Apparatus

The organization of a growing daughter ring complex is quite different from the basal complex of an adult parasite. The basal complex of growing daughters is an annulus without any clear polarity (e.g. Figure 5, t = 70–110 min). The mature basal complex in adult parasite, however, is a conical structure, closed at one end, compartmentalized, and stratified along its anterior-posterior axis (cf. Figure 1). When is the polarity and compartmentation of the basal complex established? The basal complex starts to constrict before cytokinesis and the constriction continues after cytokinesis when the daughters take over the mother's plasma membrane, thus closing the basal cap in the mature parasite (cf. Video S1) [16,17]. Because TgCentrin2 resides in only the most constricted region of the basal complex in the adult parasite (cf. Figure 1D), I investigated the relationships among the recruitment of TgCentrin2, the constriction of the basal complex and the establishment of the polarity of the basal complex, by examining TgCentrin2 localization at several different stages of cell division.

**Figure 8:**
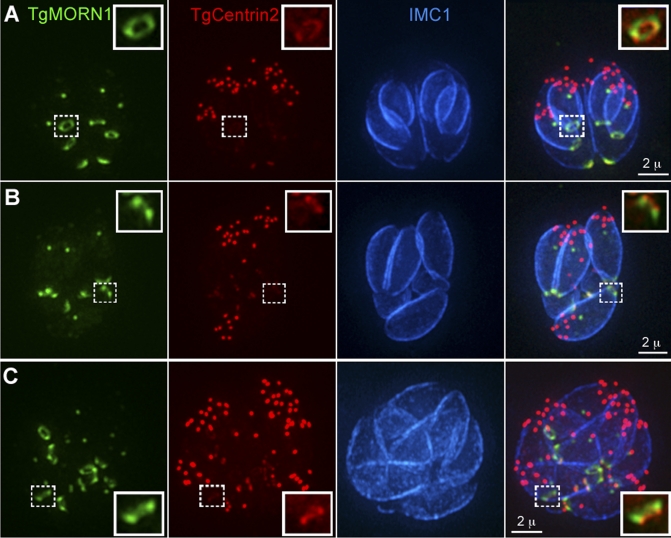
TgCentrin2 Is Recruited to the Daughter Basal Complex at the Time of Basal Complex Constriction, Which Reveals the Compartmentation and Polarity of the Basal Complex before the Closure of Its Posterior End (A) TgCentrin2 is clearly localized to a ring structure (indicated by dotted frames) in daughter parasites with a partially constricted basal complex. (B) TgCentrin2 basal labeling (indicated by dotted frames) in the daughter is pronounced during cytokinesis, when the daughters start to take over mother's plasma membrane. (C) A TgCentrin2 ring (indicated by dotted frames) is also present in parasites that have just finished cytokinesis, but whose basal complexes are not yet fully closed. Green, mCherryFP-TgMORN1 (pseudo-color); red, EGFP-TgCentrin2 (pseudo-color); blue, anti-IMC1 antibody detected by Alexa350-anti-mouse IgG. Similar exposure time (0.8 s/plane for Figure S3 and [A and B], and 0.6 s/plane for [C]) and the same brightness, and contrast setting were applied to the TgCentrin2 panels in Figure S3 and this figure to enable comparison of the relative level of Tgcentrin2 in the daughter basal complexes at different stages. Insets: 2× magnification of regions indicated by the dotted frames. The insets in the rightmost panels merge only TgCentrin2 and TgMORN1 images, and different brightness/contrast settings from the single channel images are applied to these panels to emphasize the difference in localization between these two proteins. All images are maximum intensity projections of deconvolved 3D stacks.

**Figure 9:**
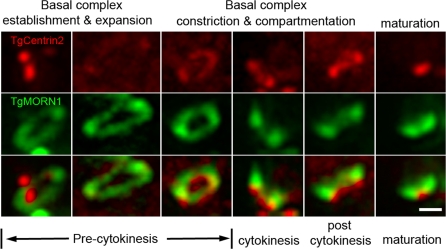
The Establishment, Construction, and Maturation of the Basal Complex throughout the Cell Cycle A collection of the enlarged insets from (left) Figure 3B; Figure S3; Figure 8A–8C and Figure 1D (right) for side-by-side comparison of morphological and molecular composition differences among basal complexes at different stages of construction and maturation. For ease of comparison, all images are oriented such that the posterior end of the parasite is closer to the bottom. Scale bar = 0.5 μm

Interestingly, although TgCentrin2 is hardly detectable in the basal ring complex earlier during cell division (cf. Figure 3B and Figure S3), it is clearly localized to the daughter basal complex as a ring at a late stage when the daughter basal complex appears to be constricted (Figure 8A). Its ring-like localization in the basal complex is also pronounced during cytokinesis when the daughters start to take over mother's plasma membrane (Figure 8B), and after cytokinesis when the basal complex is still open at both ends (Figure 8C). In all cases, the TgCentrin2 basal ring is located to the posterior of the TgMORN1 ring (Figures 8 and 9). The compartmentation and polarization of the basal complex are thus revealed upon the recruitment of TgCentrin2 prior to the closure of the basal complex.

**Figure 10:**
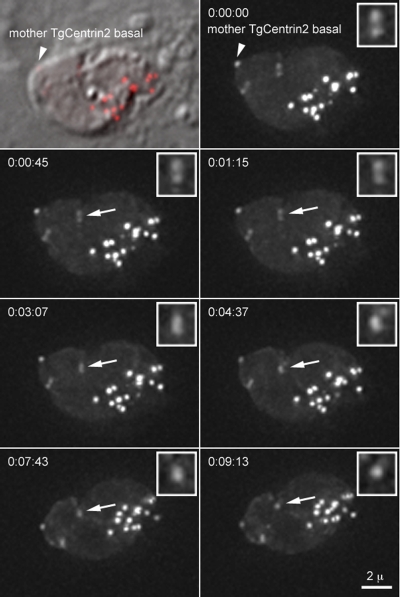
The Constriction of the Daughter TgCentrin2 Basal Ring at a Late Stage of the Cell Cycle Can Be Induced by Treatment with Calcium Ionophore A23187 (cf. Video S3) A parasite expressing EGFP-TgCentrin2 that has started its cytokinesis. EGFP-TgCentrin2 has already been recruited to the basal complex of the daughters (one of which is indicated by the arrows) at this point. Upon A23187 treatment, the EGFP-TgCentrin2 labeling clearly constricts from ∼0.9 μm (time 0) to ∼0.5 μm (time 0:09:13) in less than 10 min. The morphology of the basal complex in control parasites at a similar stage does not change when treated with 0.1% DMSO alone (unpublished data). Arrowheads, TgCentrin2 basal complex labeling in the mother cell. Insets: 2× magnification of the daughter basal complex indicated by the arrows. All images except for the DIC image are maximum intensity projections of deconvolved 3D stacks.

Like the TgMORN1 compartment, the TgCentrin2 basal compartment also undergoes significant constriction, from a ∼1.0 μm ring to a diffraction limited spot (Figures 8 and 9). Consistent with the involvement of centrin homologs in calcium sensitive contractile apparatus in other systems [22–24], the constriction of the TgCentrin2 basal compartment can be artificially induced in daughter parasites at a late stage of cell division when the intracellular calcium concentration is elevated by treatment with calcium ionophore, A23187 (Figure 10; Video S3).

## Discussion

**Figure 11:**
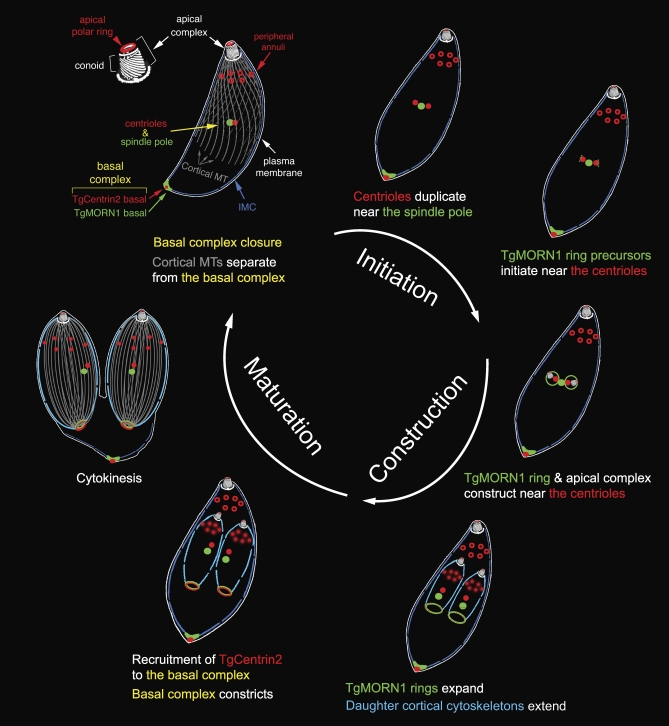
Cartoon Diagrams Summarizing the Structural Development of the Basal Complex in the Context of Other Defining Features of the T. gondii Cytoskeleton at Various Points during T. gondii Cell Cycle The cell cycle is traversed starting with an interphase adult cell at the top left and proceeding clockwise through early, mid, and late stages of daughter formation. The color scheme is such that TgMORN1 containing structures are labeled green (e.g., TgMORN1 basal compartments in the mother and daughter, spindle pole), and all TgCentrin2 containing structures are labeled red (e.g., TgCentrin2 basal compartments in the mother and daughter, centrioles, peripheral TgCentrin2 annuli, and the apical polar ring). Cortical microtubules are labeled gray. The conoid and the plasma membrane are labeled white. The daughter and mother IMCs are labeled with different shades of blue. Cortical microtubules are always present in both the mother and in daughters from early stage of daughter development, but are shown only for the daughter parasites during cytokinesis and for the interphase adult parasite.

The basal complex is a fascinating cytoskeletal organelle whose biogenesis is an integral part of parasite division during both endodyogeny and polyendodyogeny in apicomplexan parasites [16,17], including cases where triplets are formed after centriole triplication (Data not shown). In this paper, I investigated the full course of development of the basal complex of T. gondii with respect to the centriole duplication and the construction of the daughter cortical cytoskeleton (Figure 11). This paper yields new insights into the polarity establishment during cell division of T. gondii; however, it also brings to light many puzzles yet to be solved.

### What Is the Template for the *De Novo* Formation of the Basal Ring Complex?

The results in this study clearly show that although the basal ring complex later becomes the distal end of the daughter cortical cytoskeletons, it is one of the first cytoskeletal structures assembled rather than the last. In addition, the daughter cortical cytoskeleton is unlikely to provide a structural base or template for the initiation of the basal complex, as the initial construction of the basal complex is largely unaffected when the cortical microtubule construction and the structural integrity of daughter IMC complex is abolished by oryzalin treatment.

How is a macromolecular assembly like the basal complex built from scratch? Although untemplated *de novo* assembly of huge macromolecular assemblies certainly can occur (e.g., T4 phage or other large viral particles), templated construction proceeding from an inherited “seed” seems to be the rule for most large structures in eukaryotes. Interestingly, the initiation of the basal complex spatially and temporally coincides with the replication of the self-duplicating cytoskeletal organelle- the centrioles, which makes the centrioles a particularly attractive candidate for providing the structural information to initiate the *de novo* assembly of the basal complex. However, it is also clear that the centriole itself is unlikely to be continuously responsible for the maintenance of the basal ring complex, as the diameters of the rings grow up to 1 μm, much larger than the size of the centrioles while they still surround the centrioles at an early stage of cell division. Thus if the centrioles play a role in the initiation and construction of the basal ring complex, other structures associated with it probably serve as intermediary. Future high resolution EM experiments will be essential to elucidate structural connections between the centrioles and the basal ring complex.

### How Can the Elements of the Basal Ring Structure Be Retained Together?

TgMORN1 sometimes is seen to form long fibers in the cytoplasm, suggesting that this protein might have the propensity of interacting with itself and possibly form polymeric structures [16]. It is thus conceivable that the basal ring structure could be a product from a TgMORN1 polymer constrained into a ring form through its interaction with other proteins in the basal complex and/or the IMC. This, of course, is an extremely crude guess based on the scanty experimental data available. The final answer to this question will have to come from *in vitro* reconstitution experiments after we know enough about the protein composition and protein-protein interactions within the basal complex.

### What Provides the Driving Force for the Constriction of the Basal Rings?

Compared with that in daughter parasites under construction, the basal complex of fully mature parasites has constricted by ∼30%–40%. Much of the constriction occurs during the post-cytokinesis phase, as the basal complex is still a ∼1 μm ring when the cytokinesis has completed (As a reference, the diameter of the mother mitochondria is around 0.3–0.4 μm [25–27]).

What drives this constriction? Actin/myosin containing contractile rings drive the cytokinesis of mammalian cells and yeast. However, although a type XIV Myosin, TgMyoC, was found in the basal complex [28], so far available evidence does not support the involvement of the actin-myosin apparatus in basal complex constriction, as Gubbels et al. reported that cytochalasin D treatment did not seem to affect the TgMORN1 distribution [17].

A family of EF-hand containing calcium binding proteins, the centrins, underlie another type of contractile apparatus: calcium-sensitive contractile fibers associated with the algal flagella and basal body apparatus [22–24,29,30]. In this study, I found that TgCentrin2, one of the four centrin homologs in T. gondii, is recruited to a ring structure at the basal end of the daughter parasites before the onset of cytokinesis, and shrinks to a small spot at the basal tip of the adult parasites. The focused localization of TgCentrin2 at the distal portion of the basal complex also correlates with greater constriction of this region in the adult parasites. Furthermore, a treatment that elevates the intracellular calcium level induces the constriction of the TgCentrin2 basal ring. TgCentrin2 also contains multiple potential phosphorylation sites in its EF hand domains, which could be the key in regulating this contraction process, as centrin dephosphorylation accompanies calcium-flux induced centrin-fiber contraction in algae [31].

TgCentrin2, therefore, is recruited at the right time, to the right place, and possesses the right molecular characteristics to drive the closure of the basal complex at its posterior end during its maturation. Future experiments exploring conditions affecting the assembly and function of centrin contractile fiber, such as proton concentrations, and other intracellular signals will be crucial to test the role of TgCentrin2 in the basal complex constriction.

### What Drives the Distal Migration of the Daughter Basal Complex?

The basal complex migrates distally away from the apical end of the daughter parasite as the daughter cortical cytoskeleton grows. There are at least two phases to this movement. Before cytokinesis, the basal complex lies at the ends of both the daughter IMC and the cortical microtubules. After cytokinesis, however, it migrates further distally and becomes clearly separated from the cortical microtubules. Therefore although it is plausible that the directional growth of cortical microtubules drives the movement of the basal complex away from the apical pole of the growing daughter before cytokinesis [17], there is not yet any evidence to suggest a causal relationship between the basal complex distal migration and cortical microtubule growth, and the later distal movement of the basal complex during post-cytokinesis growth of the newly emerged parasites is clearly independent of microtubule growth. On the other hand, the distal migration of the basal complex **is** in synchrony with the growth of the IMC complex throughout the daughter construction, the directional growth of the IMC complex (the mechanism of which is yet to be elucidated) is therefore more likely to act as the driving force for the daughter basal complex migration.

To summarize, the daughter basal complex in T. gondii is initiated close to the duplicated centrioles, and its construction is independent of the structural integrity of the daughter cortical cytoskeleton. The daughter basal complex is a dynamic cap, whose compartmentation and polarity is revealed upon the recruitment of TgCentrin2 to its posterior end during late stages of cell division. The stage specific recruitment of TgCentrin2, its localization to the most constricted region of the basal complex, and the calcium sensitive nature of the basal complex contraction make TgCentrin2 an attractive candidate for driving the closure of the basal complex, which thus is likely to be a new centrin-based contractile apparatus. This study extends our knowledge of the origination, dynamics and coordination of the growth of distinct compartments in the daughter cytoskeletons of T. gondii. These issues are not only important for understanding, and eventually manipulating the cell biology of the apicomplexan parasites, but also of interest to the cell biology field in general, where the rules for the construction of macromolecular assemblies and polarity determination are hotly sought after.

## Materials and Methods

### Parasite culture and transfection.

T. gondii tachyzoites (strain RH) were cultivated in human foreskin fibroblast (HFF) cells, and transfected as previously described [32]. For each transfection, 1/3 of the RH parasites from a T12.5 flask culture (∼1 × 10^7^) were transfected by electroporation with 30 μg of plasmid DNA and allowed to infect a fresh monolayer of host cells.

EGFP-TgCentrin2, EGFP-TgIMC4, EGFP-TgMORN1 expressing parasites are clonal stable transgenic cell lines, and were cultured with 1μM pyrimethamine selection [16].

### Construction of fusion proteins.

pmin-mCherryFP-TgMORN1 was constructed by replacing the TgDLC/BglII-AflII fragment in pmin-mCherryFP-TgDLC with the TgMORN1 BglII-AflII fragment from pmin-EGFP-TgMORN1 [16]. pmin-mCherryFP-TgDLC was constructed by replacing the EGFP/NheI-BglII fragment in pmin-EGFP-TgDLC [16] with mCherryFP/NheI-BglII fragment from ptub-mCherryFP-EGFP. The architectures of all pmin-XFP-TgGeneX plasmids are identical.

ptub-mCherryFP-EGFP and ptub-mCherryFP-TgtubA1 were kind gifts from Dr John Murray at University of Pennsylvania.

### Light microscopy.

Fixed cells were prepared and observed as previously described [2,16,33]. Mouse monoclonal antibody anti-IMC1 was a kind gift from Dr. Gary Ward (University of Vermont) and it was detected with secondary antibody goat anti-mouse IgG Alexa350 (#A21049, Invitrogen-Molecular Probes, 1:1000 dilution). For live-cell imaging and time-lapse microscopy, parasites were inoculated into a sub-confluent layer of HFF cells grown in phenol red free DMEM (#21063, Invitrogen-Gibco) with 10% heat-inactivated bovine calf serum in a 35mm plastic dish with #1.5 glass coverslip bottom (MatTek #P35G-1.5–14-C, MatTek #P35G-1.5–20-C). After infection, the medium was changed to DMEM+1% heat-inactivated Fetal Bovine Serum (FBS). Immediately before imaging, the medium was changed to phenol red free CO2 independent medium (custom order, SKU#: RR050058, Invitrogen-Gibco) with 10%FBS and 2mM glutamine (#25030, Invitrogen-Gibco), 1mM sodium pyruvate (#11360, Invitrogen-Gibco) and 100unit/ml antibiotics and antimycotics (#1172, Invitrogen-Gibco). The dish was then equilibrated in the humidified microscope environmental chamber [34] for 1–2 hours before imaging.

3D image stacks were collected at z-increments of 0.3 μm (fixed samples), or 0.3–0.5 μm (live samples) on an Applied Precision Delta Vision workstation based on an Olympus IX-70 inverted microscope, using a 100× NA 1.35 oil immersion lens with immersion oils at refractive indexes of 1.524 (37°C, ambient humidity), 1.522 (37°C, 70% humidity in the chamber), or 1.518 (room temperature, ambient humidity). Sedat Quad- ET (#89000, Chroma Technology Corp.) and GFP/mCherry-ET (#89021, Chroma Technology Corp.) filter sets were used for all the imaging in this paper. For estimating mis-registration induced by lens chromatic aberration or optical misalignment between the GFP and mCherryFP filters, 0.2 μm Tetraspeck beads (#T7280, Invitrogen-Molecular Probes) attached to a #1.5 coverslip were mounted to a slide with a 0.12mm spacer (#S24735, Invitrogen-Molecular Probes) in dH_2_O and imaged at 37°C with the GFP/mCherry ET filter set, using 100× NA 1.35 oil immersion lens and immersion oil at refractive index of 1.522.

Deconvolved images were computed using the point-spread functions and software supplied by the manufacturer. All fluorescent images in the figures, except for the gray scale images in Figure 6 and Figure S2, are maximum intensity projections of deconvolved 3D stacks. The gray scale images in Figure 6 and Figure S2 are non-deconvolved single optical planes. The brightness and contrast of images used in the final figures were optimized for color prints.

To avoid confusion, a consistent coloring for each T. gondii protein has been adopted throughout the remainder of this paper. This pseudo-color assignment will be maintained regardless of the actual wavelength bands used for detections of fluorescence. TgMORN1 is always colored green, IMC1 is always colored blue, and other molecules are always colored red.

FRAP experiments were performed on the same imaging system equipped with a 10mW 488 laser connected to the microscope via a fiber optic. The laser was run at 50% power, using one 50 msec pulse for photobleaching. Pre and post bleaching images were collected using the Photokinetics module integrated in the Applied Precision Softworx software.

### Immuno-electron microscopy.

About 3 × 10^7^ transgenic parasites (harvested from one T12.5 flask culture) expressing EGFP-TgCentrin2 or EGFP-TgMORN1 were washed, resuspended in 15 μl DPBS (#14190, Invitrogen-Gibco) and absorbed to nickel grids (5 μl parasite suspension/grid) at room temperature for 1 hour. Parasites were then permeabilized in 0.5% TritonX-100 in DPBS for 20–25 min, and fixed for 15 min with 3.7% formaldehyde in DPBS, and washed 3 × 5 min with DPBS, followed by 10-min blocking in 5% BSA + 0.1% fish gelatin. Free aldehyde groups were blocked by incubation with 50mM glycine in DPBS for 15 min (pH 7.5) followed by 15-min incubation with 0.1% NaBH4 in DPBS. Grids were washed twice with DPBS; blocked again with 5% BSA + 0.1% fish gelatin in DPBS for 30 min; washed 2 × 5 min with incubation buffer (0.8%BSA, 0.1% fish gelatin in DPBS plus 10mM NaN3); incubated for ∼2 h with primary antibody at room temperature by inverting the grids on 20 μl drops of primary antibody (#A11122, rabbit anti-GFP polyclonal, Invitrogen-Molecular Probes, diluted 1:800 in incubation buffer); washed 6 × 5 min in incubation buffer; inverted on 15 μl drops of secondary antibody solutions (anti-rabbit IgG conjugated with 1.4 nm gold, Nanoprobes, Yaphank, New York, United States, diluted 1:160 in incubation buffer) and incubated for ∼19 h at 4 °C; then washed with incubation buffer as follows: 3 × 1 min, 2 × 10 min, 4 × 5 min; and finally washed 5 × 1 min with DPBS. The samples were then post-fixed 5 min with 1% glutaraldehyde in DPBS and washed with distilled water 3 × 5 min. Silver enhancement was carried out using the HQ silver enhancement kit (Nanoprobes) by floating grids on mixtures of the initiator, activator, and modulator for 2 min in a light-tight chamber, then washing briefly with dH_2_O once, followed by 2 × 5 min wash. Grids were negatively stained using 2% phosphotungstic acid (pH 7.0).

### Oryzalin treatment.

After taking a set of pretreatment images, 0.625 μl 10mM oryzalin diluted in 500 μl CO2 independent medium with 10% FBS (prewarmed to 37°C in the imaging chamber) was added immediately to the dish containing 2.0 ml medium on the microscope stage. The final concentration of oryzalin was 2.5 μM. Imaging of the drug treated parasites started ∼30 min after the addition of oryzalin and continued for 10–12 hours.

### A23187 treatment.

A23187 treatment experiments were conducted under both 37°C and room temperature conditions, which yielded similar results. The induction of the basal complex constriction however occurred much faster at 37°C, which made it difficult to capture the intermediate images. The result from a room temperature experiment was thus used for this paper (Figure 10). After taking a set of pretreatment images, 0.85 μl 5mM A23187 (dissolved in DMSO) diluted in 220 μl CO2 independent medium with 10% FBS was added to the dish containing 1.5 ml medium on the microscope stage, which gave a final concentration of A23187 at ∼2.5μM. After ∼3.5 minutes, additional 1.15 μl 5mM A23187 (dissolved in DMSO) diluted in 280 μl CO2 independent medium with 10% FBS was added to the dish, which gave a final concentration of A23187 at ∼5μM, and DMSO concentration at ∼0.1%. Images were taken at 15 second intervals for the first 105 seconds of acquisition and at ∼30 second intervals for the rest of the experiment. The free calcium concentration in CO2 independent medium +10%FBS was ∼3.5mM, determined by Eriochrome Black T dye assay described below.

### Eriochrome Black T dye-Ca^2+^ titration assay for determining free calcium concentration.

To 1.0 ml ammonia buffer (0.0214% NH_4_CL+ 0.73%NH_4_OH in dH2O), 5 μl Eriochrome Black Dye solution (0.25% Eriochrome Black T and 2.26% hydroxylamine hydrochloride (NH_2_OH-HCL) in dH2O) was added. 1.0 ml of CO2 independent medium +10%FBS was then added to the mixture, which turned the blue-green solution to purple. After adding 35 μl 0.1M K_2_EGTA drop-wise to the solution, the purple color turned back to blue-green due to the chelation of the free calcium by K_2_EGTA, revealing that there was ∼3.5mM free calcium in CO2 independent medium +10%FBS. The result was confirmed by titrating 1 ml 3.5mM CaCl_2_ with 0.1M K_2_EGTA using the same procedure.

## Supporting Information

Figure S1 The Shift between the Red and Green Imaging Channels Induced by the Microscope Setup Is Clearly below the Resolution Limit of the Microscope (100 KB PDF)

Figure S2 Protein Exchange Occurs in the Mature Basal Complex (203 KB PDF)

Figure S3 TgCentrin2 Is Barely Detectable in the Basal Complex of Growing Daughters before the Basal Complex Constriction (188 KB PDF)

Video S1 Time-lapse Images Showing That the TgMORN1 Ring Is Initiated around the Duplicated Centrioles This video shows a time-lapse experiment tracking the cell division of four parasites expressing EGFP-TgMORN1 (green) and mCherryFP-TgTubA1 (red). At time 0, centriole duplication (red arrows) has occurred, and in addition to TgMORN1 fluorescence in the spindle poles, there are two small extra masses of EGFP-TgMORN1 fluorescence (green arrows), one located on each side of the spindle pole, overlapping with the centriole labeling by mCherryFP-TgTubA1 (red arrows). Twenty to 30 min later, the fluorescence of mCherryFP-TgTubA1 in the centriole increases (red arrows), likely correlated with the initial assembly of the conoid in the apical complex, of which TgTubA1 is a major component. At this point, the ring-like nature of the TgMORN1 containing structure becomes apparent, and it is centered around the centriole/conoid mass (green arrows, t = 40 min). The TgMORN1 rings then undergo further expansion. At t = 60 min, the centriole/conoid assemblies move apically above the plane of the TgMORN1 ring with the extension of the cortical microtubules, and the recruitment of TgMORN1 to the apical complex becomes clear (green arrowheads). TgMORN1 rings clearly start to constrict (t = 2 h 32 min) before the onset of cytokinesis (t = 2 h 42 min). Insets: 2× magnification of regions indicated by the arrows. The images were collected at ∼10 min intervals at 37 °C. The total elapsed time for the video sequence is 2 h and 52 min. All images in the video are maximum intensity projections of deconvolved 3D stacks.
(6.5 MB MOV)

Video S2 The Initial Construction of the TgMORN1 Ring Is Likely to Be Independent of the Structural Integrity of the Daughter Cortical Cytoskeleton A parasitophorous vacuole containing eight dividing parasites expressing EGFP-TgMORN1 (green) and mCherryFP-TgTubA1 (red) treated with 2.5 μM oryzalin. This concentration of oryzalin was shown previously to inhibit the formation of the spindle and the daughter cortical microtubules, but not the centriole replication, during T. gondii cell division [9]. No daughter cortical microtubules can be detected in these parasites. The initiation (33–48 min) and construction (60–120 min) of the basal complex are not affected. Notice that the mother's conoid, cortical microtubules, and basal complex are not affected by the oryzalin treatment, and the overall shape of the mother cell remains normal (0–135 min) until the distorted daughters attempt to bud (150–165 min). As previously reported [9,17], the spindle pole fails to replicate with 2.5 μM oryzalin treatment. Images were collected at 37 °C. All images except for the first two time-points were collected at ∼15 min intervals. The time interval between the first two time-points was 33 min. The total elapsed time for the video sequence is 4 h and 45 min. All images in the video are maximum intensity projections of deconvolved 3D stacks.
(6.2 MB MOV)

Video S3 The Constriction of the TgCentrin2 Basal Compartment at a Late Stage of the Cell Cycle Can Be Induced by Calcium Ionophore A23187 A parasite expressing EGFP-TgCentrin2 that has started its cytokinesis. EGFP-TgCentrin2 has been recruited to the basal complexes of the daughters at this point (one of which is indicated by the arrow in the first frame). Upon A23187 treatment, the EGFP-TgCentrin2 labeling clearly constricts from ∼0.9 μm to ∼0.5 μm in less than 10 min. The experiment was conducted at room temperature and the interval between each image was ∼30 s. The total elapsed time for the video sequence is 10 min and 43 s. Note in some frames, such as time-points 0:02:37, 0:05:43, and 0:06:43, the upper daughter basal complex appears to be transiently elongated. This is an artifact caused by a small piece of fluorescent material that floated into the field of view and was transiently superimposed on the image of the basal complex in the projection of the 3D stack. This small object can be seen as a separate entity at time-points 0:04:07, 0:04:37, and 0:05:07. Insets: 2× magnification of the upper daughter basal complex indicated by the arrow in the first frame. All images in the video are maximum intensity projections of deconvolved 3D stacks.
(1.9 MB MOV)

### Accession Numbers

List of Tigr_final numbers for genes and proteins mentioned in the text are: TgMORN1 (583.m05359); TgCentrin2 (50.m03356); TgCentrin1 (50.m00033); T. gondii α1 –tubulin (TgTubA1; 583.m00022); TgIMC4 (44.m00031); T. gondii dynein light chain (TgDLC; 41.m01383). The sequences are available for downloading at http://www.toxodb.org/toxo/.
