# Supplementary material for: Organizational Changes of the Daughter Basal Complex during the Parasite Replication of Toxoplasma gondii
Source: PLoS Pathog. 2008 Jan 18;4(1):e10. doi: 10.1371/journal.ppat.0040010 (PMC2211554; doi:10.1371/journal.ppat.0040010)
Supplement: Supplementary file 1 [file ppat.0040010.sg001.pdf]

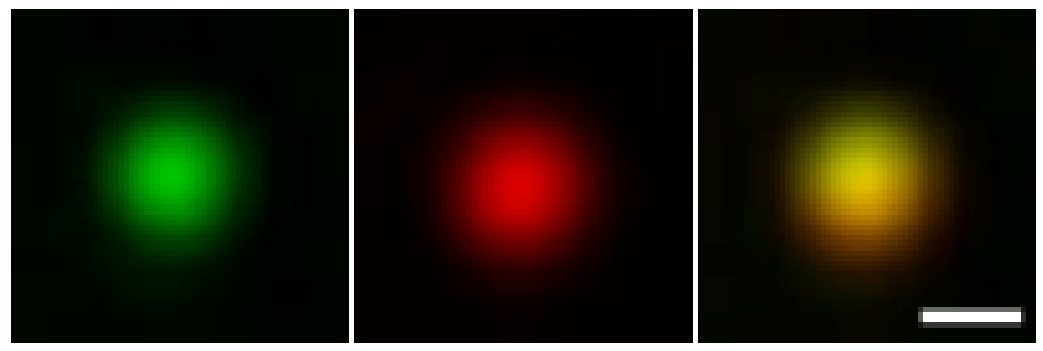

Figure S1 The shift between the red and green imaging channels induced by the microscope setup is clearly below the resolution limit of the microscope.

Images of red and green fluorescence from a multi-color 0.2 μm bead showing that the shift between the red and green channels induced by lens chromatic aberration or optical misalignment between the GFP and mCherryFP filters is clearly below the resolution limit of the microscope. Scale bar= 0.2 μm
