# Supplementary material for: Organizational Changes of the Daughter Basal Complex during the Parasite Replication of Toxoplasma gondii
Source: PLoS Pathog. 2008 Jan 18;4(1):e10. doi: 10.1371/journal.ppat.0040010 (PMC2211554; doi:10.1371/journal.ppat.0040010)
Supplement: Supplementary file 2 [file ppat.0040010.sg002.pdf]

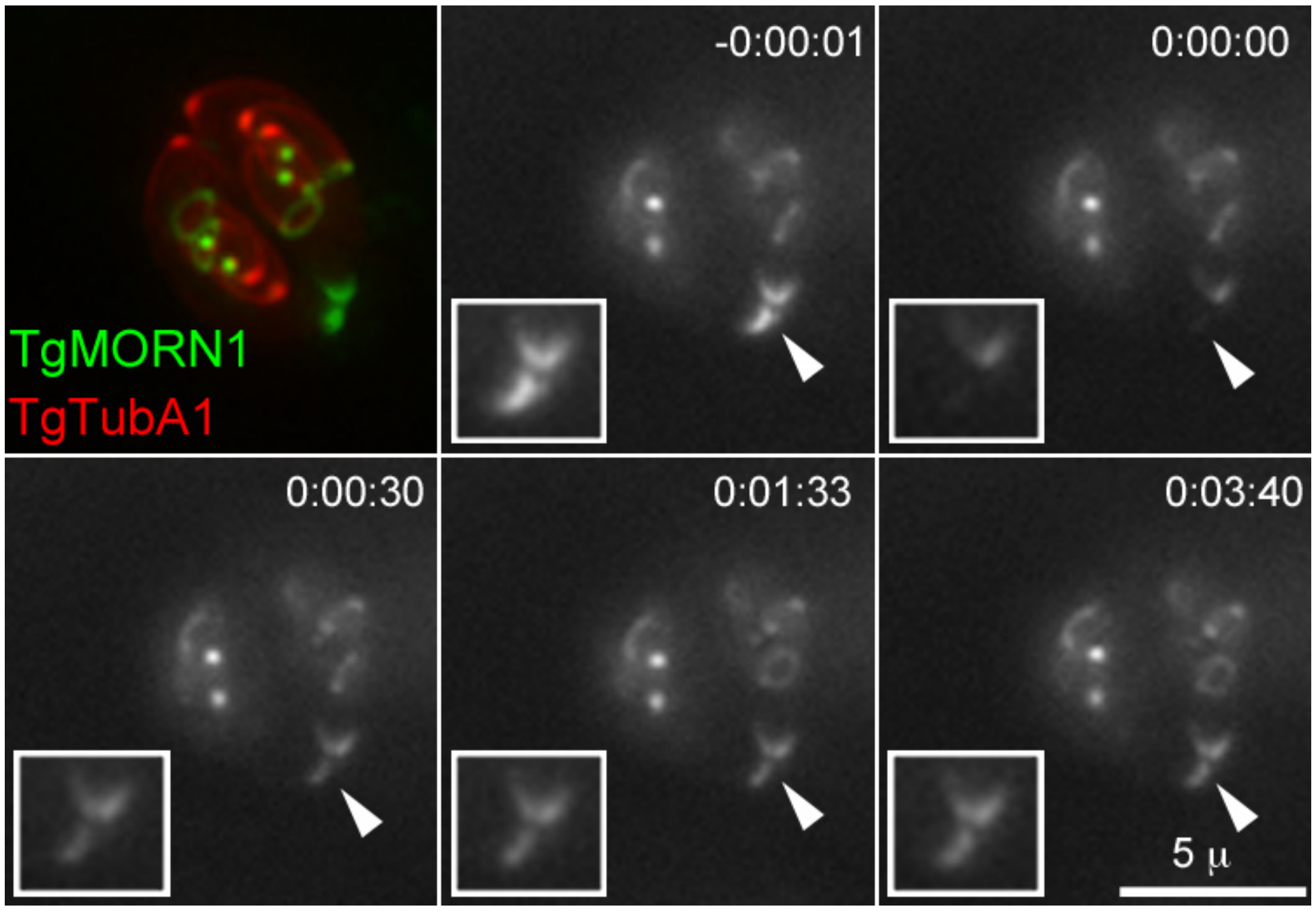

Figure S2 Protein exchange occurs in the mature basal complex.
Time-lapse experiment tracking the exchange of EGFP-TgMORN1 in the basal complexes of adult parasites using FRAP. The fluorescence of EGFP-TgMORN1 in the mature basal complexes of two dividing parasites was partially bleached (*arrowheads*, insets) at time 0. The recovery started at ~11 sec, but only partially completed over the course of 220 seconds. Insets: 1.5x magnification of regions indicated by the arrowheads.
The merged image of EGFP-TgMORN1 (green) and mCherryFP-TgTubA1 (red) is the maximum intensity projection of a deconvolved 3-D stack. All the gray scale images are non-deconvolved single optical planes.
