# Supplementary material for: Organizational Changes of the Daughter Basal Complex during the Parasite Replication of Toxoplasma gondii
Source: PLoS Pathog. 2008 Jan 18;4(1):e10. doi: 10.1371/journal.ppat.0040010 (PMC2211554; doi:10.1371/journal.ppat.0040010)
Supplement: Supplementary file 3 [file ppat.0040010.sg003.pdf]

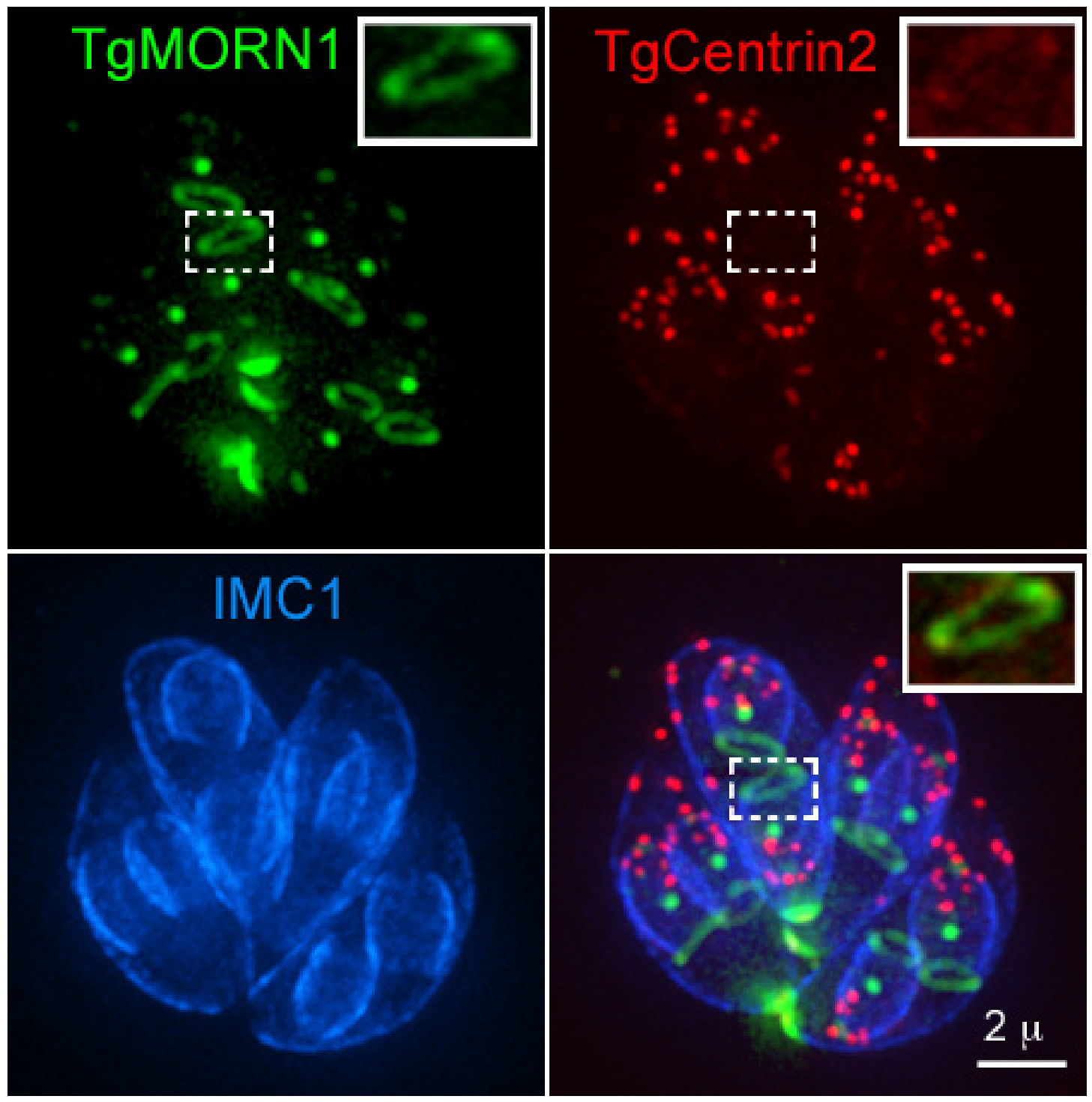

Figure S3 TgCentrin2 is barely detectable in the basal complex of growing daughters before the basal complex constriction

Fluorescent images illustrate the distribution of mCherryFP-TgMORN1 (pseudo-colored green), EGFP-TgCentrin2 (pseudo-colored red) and the IMC (blue, anti-IMC1 labeling detected by Alexa350-antimouse IgG) in 4 dividing parasites in the same parasitophorous vacuole. Very little TgCentrin2 fluorescence is seen in the TgMORN1-containing basal ring complexes (indicated by dotted frames) at this stage of daughter development.

Insets: 2x magnification of regions indicated by the dotted frames. The inset in the rightmost panel merges only TgCentrin2 and TgMORN1 images.

All images are maximum intensity projections of deconvolved 3-D stacks.
